# Sensitivity and Resistance of Parasitic Mites (*Varroa destructor*, *Tropilaelaps* spp. and *Acarapis woodi*) Against Amitraz and Amitraz-Based Product Treatment: A Systematic Review

**DOI:** 10.3390/insects16030234

**Published:** 2025-02-20

**Authors:** Michela Bertola, Franco Mutinelli

**Affiliations:** NRL for Honey Bee Health, Istituto Zooprofilattico Sperimentale delle Venezie, 35020 Legnaro, PD, Italy; fmutinelli@izsvenezie.it

**Keywords:** amitraz, mite resistance, resistance ratio, resistance index, apiculture

## Abstract

Resistance to amitraz in *Varroa destructor* mites poses a significant challenge to global beekeeping, leading to the declining efficacy of treatments and increased colony losses. This study aims to comprehensively map, characterize, and analyze the status of amitraz efficacy and resistance in Varroa and other parasitic mites such as *Tropilaelaps* spp. and *Acarapis woodi*. A systematic review, following PRISMA 2020 guidelines, examined 74 studies, revealing substantial variability in experimental protocols, mite origins, and environmental factors, all of which impacted toxicity assessments. These findings highlight the urgent need for standardized methodologies to ensure consistency and reliability. Resistance ratios (RR) and indices (RI) showed significant geographical variation, reflecting localized resistance development. Laboratory studies highlighted inconsistencies in detecting resistance, underscoring the importance of combining bioassays, molecular diagnostics, and field efficacy tests. Understanding the genetic and physiological mechanisms driving amitraz resistance, as well as their prevalence, is vital to devising sustainable management strategies. Establishing national monitoring programs and revising testing protocols are pivotal steps toward ensuring the continued effectiveness of acaricides. These measures, combined with coordinated efforts by researchers, beekeepers, and policymakers, are essential to safeguarding global honey bee populations and supporting the long-term sustainability of apiculture.

## Simple Summary

Amitraz resistance in *Varroa destructor* mites threatens global beekeeping by reducing treatment efficacy and increasing colony losses. This study reviews 74 publications to map and analyze amitraz resistance in Varroa and other parasitic mites, such as *Tropilaelaps* spp. and *Acarapis woodi*. The findings reveal significant variability in resistance levels across regions, driven by inconsistent experimental protocols and environmental factors. Integrating bioassays, molecular diagnostics, and field tests is critical for accurate resistance detection. Standardized methodologies, national monitoring programs, and updated testing protocols are essential to managing amitraz resistance effectively, ensuring sustainable Varroa control and protecting global honey bee populations.

## 1. Introduction

European honey bees (*Apis mellifera*) play a crucial ecological and economic role through their pollination of both natural ecosystems and agricultural crops [1,2], as well as their importance in beekeeping [3].

However, managed honey bee colonies have been facing significant losses due to a complex combination of factors [4,5,6,7,8]. Human activities, including habitat loss, the available nectar and pollen sources pollution, and climate change (climate and human land use changes), have severely degraded bee environments [9,10]. Additionally, chronic exposure to agrochemicals, parasite infestations, the emergence of new pathogens and predators, and inter-seasonal weather fluctuations further exacerbate the situation [11,12].

One of the primary contributors to colony losses is the infestation of hives by *Varroa destructor*, a parasitic mite that originated in Asia and has spread globally since the 1970s. This mite is now recognized as a major pathogenic agent in bee colonies [13,14,15].

Varroosis is the consequence of a high mite infestation rate in colonies [16], with Varroa mites acting as carriers and amplifiers of viruses that devastate colonies and play a key role in the overwintering losses of honey bee colonies [9,17,18,19].

A great variety of integrated pest management (IPM) strategies are used by beekeepers to maintain low *V. destructor* infestation and regard both the prevention and control of the mite. Many control methods (cultural, biological, mechanical, and chemical) are used to reduce Varroa mite populations [20,21], with chemical control often being the most effective and economical measure [22,23].

Miticide applications are generally considered the most reliable and effective method for managing Varroa mites. However, their use can pose several challenges, including the potential for leaving residues in hive products [24,25], causing toxicity to honey bees [26] and contributing to the development of resistant Varroa populations [27,28,29]. The widespread use of miticides over the years has exerted strong selective pressure on mite populations, leading to the emergence of resistant populations worldwide [30] that can survive doses or concentrations of toxic substances that previously caused high mortality rates [31]. Furthermore, the repeated, persistent, and often exclusive application of chemical miticides—whether through inconsistent use contrary to labeled instructions, lack of rotation, or the application of unregulated homemade preparations—has significantly contributed to the development of pharmacoresistance and widespread control failure [32]. This resistance initiates a vicious cycle where reduced sensitivity to miticides leads to inappropriate use and repeated over-application, leaving behind a resistant reproductive majority as susceptible mites are eliminated [13,29,33,34,35,36].

Indeed, miticide treatment efficacy depends on multiple factors, including both pharmacological and biological parameters of both the mite and the honey bee. For example, the timing of treatments, strictly connected to both the brood level and the miticide pharmacokinetics (i.e., amount of active ingredients released daily, treatment duration), also play a crucial role in determining treatment success.

The persistence of resistant mites, even when miticide treatments are correctly applied and product rotation is implemented to minimize acaricidal pressure, can be attributed to various factors [25,37].

Contributing factors include bees robbing honey from weak or dying hives already infested with resistant mites, the introduction of infested packaged bees or queens from other regions, and drifting bees, which can quickly spread phoretic mites in large apiaries [29,38].

Resistance in mites refers to their ability to survive exposure to acaricides, often due to genetic or metabolic adaptations [39]. These mechanisms may evolve over time, potentially leading to cross-resistance to multiple active ingredients [38]. Target-site mutations in molecular targets, such as the genetic modification of voltage-sensitive sodium channels (VGSC), represent a potential genetic resistance mechanism [35,36,40,41,42].

Additional resistance mechanisms in Varroa include metabolic detoxification, where changes in the expression of detoxification enzymes enable the mite to enhance its metabolism, effectively degrading the acaricide. In this case, resistance can be assessed through biochemical assays targeting general esterases, cytochrome P450 monooxygenase, and glutathione-S-transferase [22,38,43,44,45,46].

Increased tolerance of mites to various active substances can be identified through laboratory assays or inferred from reduced field efficacy [22,47]. Field trials, influenced by factors such as Varroa mite resistance to acaricides, initial infestation levels, brood quantity, and reinfestation rates [48,49], are also affected by climate change and seasonal variability, as *V. destructor* populations fluctuate with brood availability [19,50]. The presence of resistant mites is likely the primary cause of the failure of previously effective chemical treatments, and confirming resistance requires toxicological assays, biochemical tests (such as esterase, cytochrome P450 monooxygenase, and glutathione-S-transferase), or genetic analysis using mites collected from various apiaries across different regions [22]. To standardize the methodology for determining amitraz resistance, the lowest concentration that results in complete mortality and the highest concentration with no effect must be estimated. Within this range, five intermediate concentrations should be selected and spaced logarithmically. These concentrations are then tested on Varroa mites, using at least five replicates, each consisting of ten mature females per dilution [30,31].

A key metric for evaluating resistance is the lethal concentration required to achieve 50% mortality (LC50) in a known susceptible population, which serves as a reference point for comparison. The toxicological assay for determining amitraz resistance involves directly exposing mites to acaricidal products and calculating the lethal concentration (LC50) that kills 50% of individuals in the target mite population [30], then comparing it to the LC50 of a susceptible reference population from the same area.

Mortality data should be analyzed using Probit analysis to determine the slopes and intercepts of concentration–response curves, with mortality as the dependent variable and concentration as the independent variable [51]. While the slopes of concentration–response curves are typically consistent across populations, a reduced slope in the concentration–response curve of a population exhibiting high resistance, compared to a susceptible population, indicates a significant level of resistance [31].

Mortality values should be adjusted based on natural mortality observed in control groups to provide a more accurate measure of treatment efficacy. Different formulas, such as Abbott’s and Schneider-Orelli’s, can be applied interchangeably. However, the Abbott formula is more commonly used, while the Schneider-Orelli formula explicitly emphasizes survival rates [52,53].

Additionally, the lethal concentration required to kill 90% or 95% of the population (LC90 or LC95) can also be used; however, the difference compared to the reference population will be even more pronounced [30].

Resistance in mite populations can be expressed using the Resistance Ratio (RR) or Resistance Index (RI), which are different terms for the same calculation [27,29,30,51]. Both involve determining the ratio of the lethal concentration (LC50) required to kill 50% of the mites in a resistant population compared to the LC50 of a susceptible reference population. A higher RR or RI indicates a greater level of resistance in the population.

Usually, for resistance assessment, mature female mites collected from capped brood should be used, but no significant differences were found among mites from different brood stages (excluding pupae with dark bodies, when newly molted adult mites may be present). Therefore, the results of assays carried out on Varroa females from different brood stages could be combined, and in some later tests, when the number of mites was too low to assay them separately, mites taken from different stages could be pooled [30,34].

While mites collected from adult bees can also be used, they should be avoided at the beginning of autumn due to their altered physiological condition, which includes reduced weight, increased water loss, and shorter lifespan. This different physiological condition will lead to different and rather variable response and an increased natural mortality of mites during the resistance test [30].

Alternatively, assessing mite resistance is possible through biochemical or genetic analyses comparing the target mite population with a susceptible stock population [54].

The acaricide resistance in *V. destructor* is well documented for tau-fluvalinate and coumaphos and has led to a decrease in treatment efficacy shortly after their introduction [28,31,55,56,57,58,59].

For many years, amitraz withstood any signs of resistance development in most countries, despite being used for Varroa control, which suggests that the selection pressure should be lower than that exerted by the other acaricides [19,21,31,60,61]. Many veterinary medicinal products containing amitraz as an active ingredient are used in Varroa control [21].

Consistent field reports of reduced amitraz efficacy are particularly concerning, especially given the beekeeping industry’s reliance on this compound for *V. destructor* control. The increasing prevalence of amitraz-resistant Varroa populations has raised significant alarm among beekeepers, veterinarians, bee inspectors, and honey bee health researchers [62].

The evolution of resistance to amitraz is a serious problem not only due to its use in beekeeping for controlling mites and lice, but also for combating parasites in livestock and pets [61,63].

Therefore, the aim of this study is to map, characterize, and analyze the current status of *V. destructor* mite sensitivity and resistance to amitraz and amitraz-based products worldwide. This review will specifically focus on laboratory bioassays (toxicological, metabolic, or genetic assays) because they could reliably inform onthe degree of mite resistance to an active substance, and from this the treatment efficacy in the field could be speculated.

This study also investigates the sensitivity and resistance of *Tropilaelaps* spp. and the honey bee tracheal mite, *Acarapis woodi*, to amitraz, given their significant impact on bee health. While *A. woodi* is currently found at very low frequencies in the Americas and EU, it continues to spread and grow in regions such as Japan, making constant vigilance essential, as resistance could emerge over time [64]. In contrast, *Tropilaelaps* spp. requires close monitoring and more research due to its recent expansion into Europe, emphasizing the growing need for proactive measures to prevent its further spread and reduce its potential risks to apiculture [65,66].

## 2. Materials and Methods

### 2.1. Review Question, Eligibility Criteria, Information Sources, and Search Strategies

This systematic literature review was conducted in accordance with PRISMA 2020 guidelines [67]. The central research question was as follows: “What is the current status of *V. destructor*, *Tropilaelaps* spp., and *A. woodi* mites’ sensitivity and resistance to amitraz and amitraz-based products based on toxicological laboratory studies”? This review focuses on three key elements: (i) population: parasitic mites of honey bees (*V. destructor*, *Tropilaelaps* spp. and *A. woodi*); (ii) intervention: amitraz and amitraz-based products; (iii) outcome: decreased sensitivity or confirmed resistance in toxicological laboratory studies. To ensure comprehensive coverage, a variety of relevant synonyms were used in the full-text search, with detailed search strategies outlined in Supplementary File S1. For example, both names, *V. destructor* and *V. jacobsoni*, were used in the search strings to ensure that no important information was missed [68].

We included peer-reviewed studies indexed in PUBMED and the Web of Science Core Collection up to 3 January 2025.

The following eligibility criteria were applied: (i) the study must be in English language with full-text availability; (ii) it must report primary research data; (iii) it has to involve *V. destructor* and/or *Tropilaelaps* spp. and/or *A. woodi*; (iv) parasitic mites have to be collected from *A. mellifera* colonies; (v) the study had to report data on amitraz or amitraz-based products; and (vi) the results of the study must involve a reduction in efficacy/increase in sensitivity or an effective resistance assessment under laboratory conditions.

The screening process was conducted by two reviewers (MB and FM), who firstly classified all of the studies from the initial literature search based on their titles and abstracts. When abstracts were unclear or lacked sufficient details, or when there was any uncertainty about the reported data, the study was included for full-text evaluation. Each record was independently assessed by the reviewers using the pre-established eligibility criteria.

After retrieving the full texts, the selected studies were categorized for separate analysis: (i) loss of efficacy in laboratory bioassays involving direct exposure of mites to acaricidal products, and (ii) resistance assessments, including toxicological laboratory assays and metabolic or genetic analyses comparing sensitive and resistant populations (Supplementary File S2).

Additional data relevant to amitraz efficacy and resistance were extracted, such as the Varroa life cycle stage (i.e., sampled from adult bees or brood), seasonality, and amitraz or amitraz-based product details (amount, exposure and incubation duration, environmental parameters, …), and are summarized in Supplementary File S3. The most significant data categories were synthesized in tables by us and are discussed throughout the text.

### 2.2. Quality Assessment

A quality assessment framework was developed to address the qualitative aspects of the efficacy test and resistance assessment studies. Commune key factors included the following: (i) timing of mite sampling during the year; (ii) number of mites per group and replicates; (iii) environmental parameters during the test (temperature and relative humidity); (iv) the response used to categorize a mite as dead; and (v) correction for natural mortality.

Each study was evaluated based on these criteria, with a scoring system assigning 1 point for each positive response and 0 for negative responses. This provided a final quality score for each study, ranging from a maximum of 5 (high quality) to a minimum of 0 (low quality). This systematic approach ensures a robust and standardized evaluation of methodologies across studies (Supplementary File S4).

## 3. Results

### 3.1. Study Selection

After screening 206 studies, 74 were selected for inclusion. The PRISMA flowchart (Supplementary File S2) outlines the literature search and screening process. Additionally, three papers not retrieved through the primary research strategy were included based on the authors’ expertise in the field (other source).

**Figure 1:**
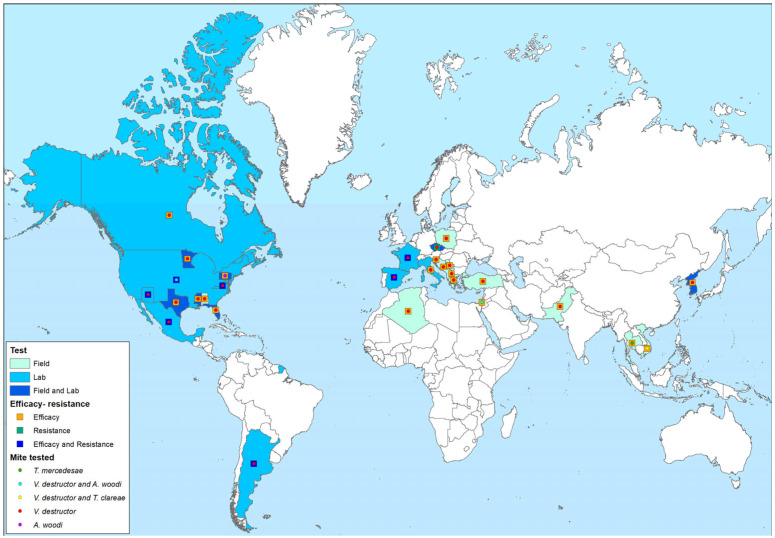
Global distribution on tested mites and type of test conducted.

The majority of the elected studies (72/77) involve *V. destructor* (named *V. jacobsoni* in [28,69,70,71,72,73,74]) (Figure 1, Table 1, Supplementary File S3), 3 field experiments have been conducted on *A. woodi* [75,76,77,78], and only 1 study has investigated the toxicological impact of amitraz on this mite both under laboratory and field conditions [78]. There is a significant gap in the literature on *Tropilaelaps* spp., with only one field study on *T. mercedesae* reporting no effect of amitraz [79]. Additionally, Woyke [70] conducted a study investigating the effects of amitraz during the co-infestation of *T. clareae* and *V. destructor*, although its findings are limited in scope. Further research is needed to better understand the impact of amitraz on *Tropilaelaps* spp. across different species and infestation levels.

Research on amitraz efficacy against Varroa mites has encompassed both laboratory and field evaluations, which are often combined within the same study to provide a more comprehensive assessment. To date, 27 studies have assessed amitraz efficacy against Varroa mites using laboratory tests, while 42 have conducted tests under field conditions (Figure 1, Table 1, Supplementary File S3). Additionally, resistance assessment evaluations for amitraz, including its quantitative determination using laboratory toxicological assays or investigations of its metabolic or genetic mechanisms, have been conducted in 12 studies.

These data come from a wide range of countries across the globe, reflecting the global interest and effort to understand and manage the *V. destructor* mite and its impact on honey bee colonies. The global distribution of tests is categorized by test type and mite species examined. This summary includes both laboratory and field trials involving amitraz and amitraz-based products, as detailed in Table 1 and illustrated in Figure 1.

The subsequent analysis focuses exclusively on laboratory resistance assays. While field tests were mapped, they are not further analyzed due to their high variability in protocols and procedures (Supplementary File S3). As emphasized by Gregorc et al. [48] and Almecjia et al. [49], field conditions introduce environmental variables, apiary management practices, and the presence of other pests and diseases, all of which can complicate the interpretation of mite resistance. These factors are often difficult to control or fully quantify, making it challenging to attribute treatment failure solely to resistance. All experimental variables are thoroughly discussed, with key results summarized in tables and the complete dataset provided in Supplementary File S3.

### 3.2. Experimental Protocol During Laboratory Assay

#### 3.2.1. Mite Origin and Pre-Bioassay Condition

All of the varroa mites tested came from *A. mellifera* colonies. Only two bee subspecies were explicitly mentioned: *A. m. iberiensis* [82] and *A. m. ligustica* [48,92]. The mite populations were either from the same region but collected in different years [29,73,128,129], or were from different regions but sampled concurrently [31,51,62,97]. The source of the mites varied, with some studies obtaining them from a single colony from one apiary [49,94], a single apiary (more colonies from one apiary) [32,33,81,83,84,85,89,90,92], or multiple apiaries (more colonies from more apiaries). Data from different apiaries could be aggregated or maintained as divided and then compared (Supplementary File S3).

A precisely described sampling procedure and clear group composition are essential for amitraz assessments, requiring a detailed specification of how Varroa mite groups were formed at both the apiary and colony levels.

The level of mite infestation in the colonies or apiaries was detailed in a few studies [82,83,85], as well as the quantity or weight of the sampled infested bees [31,81,82,83,93].

Regarding acaricide treatments, some studies described them based on the active ingredient used, particularly in cases of treatment failure, or by the duration without treatment, which ranged from a few months to instances where colonies were never treated. Typically, the investigated populations had a history of amitraz application [31,51,62] or reported amitraz therapeutic failures [29]. In contrast, the susceptible populations originate from colonies where amitraz had not been used for one year [62] or had never been used [31,97].

Additionally, while 29 out of 33 studies mentioned the year the tests were conducted, only 22 specified the months, or at least the season, when mites were collected. Given the seasonal physiological changes in both mites and bees, specifying the temporal collection period is essential for accurately assessing treatment efficacy. Furthermore, including both temporal and geographical coordinates is vital to enable meaningful comparisons with future studies conducted under similar or different conditions.

Mites can be collected from infested bees or brood in the field and transported to the laboratory, or infested bees and brood frames can be collected and brought to the laboratory, where they were incubated before testing [38,49,80,86,92,97]. Milani [30] observed that mites collected from adult bees exhibited greater variation in mortality compared to those collected from pupae, a finding later confirmed by Goodwin et al. [94], who noted that collecting mature females from adult bees is preferable for comparison, particularly when access to the entire colony is limited or unavailable. Some studies collected mites from brood (both drone and worker) by uncapping pupae, while others sampled mites from honey bees of mixed ages, workers, or nurse bees, using CO_2_ or sugar shake techniques. Almecjia et al. [49] is the only study that highlights how different mite collection methods could significantly impact mite mortality.

Maggi et al. [33] did not differentiate between brood stages, aligning with Milani [30], who suggested that brood stage does not significantly affect test variability. In contrast, Higes et al. [61] and Santiago et al. [92] specifically collected mites from pupae, highlighting methodological differences. The “decapping and brush” method, although labor-intensive, was noted for minimizing mite trauma compared to techniques like the sugar roll, which involves additional handling, including a final water rinse, but it is the less destructive method [33,88,89,90].

Pre-bioassay conditions, which are critical for maintaining the physiological state of the mites before testing, were detailed in 17 papers (Supplementary File S3). These descriptions included aspects such as mite handling, environmental conditions, transport duration, and preparation protocols prior to the assays (maintenance of mites or infested bees under optimal conditions), providing valuable context for understanding the reliability and reproducibility of the results.

Bava et al. [87] and Castagna et al. [88] also outlined mite selection criteria, excluding mites that appeared freshly molted, weak, or deformed to ensure consistent bioassay results. This careful selection minimized potential variability due to differing mite conditions.

#### 3.2.2. Bioassay Condition and Setup

The assessment of amitraz efficacy in *V. destructor* was predominantly conducted through contact toxicity assays (31 out of 33), and two studies utilized topical application through microapplication [83,92] and/or the spray method [92]. Contact assays were designed to test mites’ exposure to treated surfaces, using systems such as Petri dishes, mason jars, apiarium (an 800 mL polypropylene plastic cage holding 100–150 bees) [84], glass vials, and various plastic containers. Three experiment protocols involved spraying the active ingredient through the Potter–Burgerjon tower, which simulates field applications [92,128,129] (Supplementary File S3).

Amitraz powder was tested in 20 studies; the powder was either undiluted or mixed with solvents such as hexane, acetone, or ethanol and applied topically or by surface contact in various containers. Veterinary medicinal products like Apitraz^®^ and Apivar^®^ were also evaluated. Apitraz^®^ strips (500 mg a.i.) were tested in Petri dishes and mason jars [54,61,82,95], while Apivar^®^ strips were used in multiple experimental setups, including Petri dish, laboratory cages, and apiarium cages [31,32,48,54,73,85,91,93]. Other formulations, such as Taktic^®^, were applied in Santiago et al. [92] and Rodriguez-Dehaibes et al. [128,129]. Mitaban^®^ was tested only once by Baherini et al. [83].

Experimental conditions, including temperature and humidity, also differed significantly, ranging from 22 °C to 35 °C and from 0% to 90% humidity, with some studies not specifying these details. If adult bees are used in the tests, higher temperatures can increase the rate at which Varroa mites fall off adult honey bees, with this effect being more pronounced in stocks selected for high grooming behavior. In contrast, the background levels of Varroa dislodging under control conditions at room temperature (22–24 °C) are very low [96]. Exposure times (contact time between mites and the a.i.) ranged from one hour to 48 h, while incubation periods—the time between exposure and efficacy assessment—were reported in only few studies [61,83,84]. Only one study reported differing temperatures between the exposure period (20 °C) and the incubation period (35 °C) [94]. Typically, the mites remained in the test container after the miticide was removed; however, in Goodwin et al. [94], exposed mites were placed in a clean Petri dish following the exposure period.

Either mites were tested alone (20 out of 33 studies) or alongside larvae, pupae, or adult bees, which could be introduced after either exposure or several hours later [28]. It was crucial that the bees used in the assays were minimally infested with Varroa (less than 1% infestation), as specified by Baherini et al. [84,85]. In some studies, bees were fed sugar cubes during the tests [82,85,128], while others did not provide additional nutrition.

The evaluations of mite mortality were conducted at different intervals, with some studies measuring efficacy immediately after exposure and others extending up to 24 h post-exposure (Supplementary File S3).

Key factors influencing the outcomes included the type of contact (direct or indirect), the size of mite groups, the number of replicates, environmental conditions, and the presence of a nutrition source for mites during the assays. These variables underscore the complexity of designing standardized bioassays and highlight the importance of consistent reporting for accurate comparisons across different studies.

#### 3.2.3. Data Correction and Elaboration

The criteria for determining mite mortality varied significantly among studies, reflecting a lack of uniformity in assessment methods. In Higes et al. [61], mites were considered dead if they showed no movement when gently probed with a fine paintbrush after incubation, while in Lee et al. [90] and Bava et al. [87], Varroa mites showing complete immobility or little motion when touched with a probe were considered dead. According to Roth et al. [22], a mite was considered dead if it exhibited a lack of movement when probed, slow or minimal leg movements, or an inability to walk.

Kamler et al. [51] defined a mite as dead if it failed to escape, including those exhibiting tremors, by the end of the experiment, while mites that successfully exited the designated circle were recorded as survivors. This methodology was reaffirmed by Marsky et al. [97], emphasizing the mode of action of amitraz (e.g., amitraz targets the mites’ octopamine receptors, disrupting synaptic transmission and causing paralysis). This paralysis is sub-lethal, with death occurring indirectly due to starvation resulting from the inability to move or feed. Consequently, mites that remained paralyzed and immobile after 24 h were categorized as dead, aligning with the earlier criteria.

Since Varroa mites are obligate parasites of bees and cannot survive for long after detaching from their host, their time of detachment can be reasonably approximated as their time of death [73].

This approach has been used by Gregorc et al. [48], Rinkevich et al. [31], Vu et al. [93], and Morfin et al. [91], and mortality was assessed by collecting mites that had fallen to the bottom of a cage insert following treatment.

Baherini et al. [85] makes a distinction between mortality rate (based on the dead mite counts) and knockdown rate, defined as a percentage of the sum of all mites (both live and dead) found outside the cluster during both assessment periods.

In total, 9 out of 26 studies accounted for natural mortality when correcting their mortality data. As highlighted by Goodwin [94], natural mortality is a critical factor that should always be considered, as it can vary significantly between trials conducted in early versus late winter. Control groups are essential not only for identifying procedural errors, but also for accurately quantifying natural mortality, ensuring the reliability of the results.

### 3.3. Results and Data Interpretation

The doses and corresponding mortality rates for the selected articles are presented in Table 2.

### 3.4. Amitraz Resistance Assessment

A total of 12 studies quantitatively, metabolically, or genetically assessed the resistance of various *V. destructor* populations to amitraz or amitraz-based products such as Apivar^®^, Apitraz^®^, and Taktic^®^ (see Figure 1, Table 3, and Supplementary File S2). During laboratory resistance assessments, the discriminant concentrations (LC50) established in susceptible populations were used (*n* = 6) to quantitatively evaluate amitraz resistance in other mite populations under investigation. Only Kamler et al. [51] calculated LC50, LC90, and LC95 values.

In some cases [22,38], previously published discriminant concentration values were employed as reference points in new experimental setups. Resistance ratios (RR) were calculated in two studies [31,51], while resistance indices (RI) were utilized in three studies [29,128,129]. Additionally, median lethal time (MLT) was applied in one study [73].

To explore resistance mechanisms, metabolic assays were performed in two studies [22,38], while, to date, four studies were conducted [62,95,96,97] to investigate the genetic basis of amitraz resistance.

Notably, no evidence of amitraz resistance has been reported for other parasitic mite species such as *A. woodi* or *Tropilaelaps* spp., underscoring the specificity of resistance concerns to *V. destructor*.

#### 3.4.1. Quantitative Resistance Calculation

Rodriguez-Dehaibes et al. [128,129] adopted the methodology used by Santiago et al. [92] to track the evolution of amitraz resistance in Mexico. They determined the RI based on the baseline value in Santiago et al. [92] to assess the development in resistance through the years.

In Argentina, Maggi et al. [29] conducted a study similar to their previous work in 2008 [33], comparing the results and calculating the RI for amitraz resistance.

The RR between mite populations in different areas during the same time has been calculated in the USA and Czechia by Rinkevich et al. [31] and Kamler et al. [51], respectively.

Only one study [31] has specifically analyzed the relationship between amitraz and Apivar^®^, an amitraz-based product. In this study, both the efficacy and amitraz resistance and Apivar^®^ efficacy (tested in the field and referred to as the “Pettis’s test” [59]) were examined together. The results from in vitro amitraz bioassays show that increased resistance ratios are correlated with reduced Apivar^®^ efficacy, demonstrating bona fide cases of Varroa control failures due to amitraz resistance. Specifically, Varroa populations with an amitraz RR) greater than 10-fold and an Apivar^®^ efficacy of less than 80% can be classified as functionally resistant to amitraz.

We have considered the Mean Lethal Time (MLT) method used in Mathieu et al. [73], which measures the time required to kill mites exposed to amitraz, because it quantitatively provides a value that can be used for comparison in future studies. Mathieu’s results show increased MLT values over time, which may suggest a decline in product effectiveness.

In Table 3 is reported the variability in amitraz resistance assessments across different geographic locations and time periods. The data demonstrate a wide range of resistance indices (RR or RI), indicating diverse levels of susceptibility among Varroa mite populations. The studies from Kamler et al. [51] and Maggi et al. [29] report moderate to high resistance indexes, showing significant variation even within the same regions. Rinkevich et al. [31] presents an extensive dataset with highly variable resistance ratios, ranging from 1.7 to 22.5, underscoring the potential for localized resistance development in USA Varroa populations. The findings by Rodriguez-Dehaibes et al. [128,129] reveal high resistance indexes over time (RI: up to 26.66).

#### 3.4.2. Genetic Resistance

Amitraz mimics the neurotransmitters octopamine and tyramine by binding to their receptors (involved in recognizing extracellular messengers, transducing signals to the cytosol, and mediating the cellular responses necessary for the normal physiological functions of organisms), thereby blocking their function and leading to hyperactivity, leg waving, detachment behavior, and inhibited reproduction [62].

Two amino acid substitutions (N87S and Y215H) in the β2-adrenergic-like octopamine receptor (Octβ2R), the target site of amitraz, have been potentially associated with amitraz treatment failure events in France and the United States [62]. In Spain, Hernandez-Rodriguez et al. [95] investigated a new point mutation (F290L) in the Octβ2R receptor associated with amitraz resistance both in laboratory and field conditions, finding (i) a decrease in susceptibility to amitraz in mites previously exposed to continuous amitraz treatments and (ii) that the frequency of L290 mutated alleles increased after consecutive treatments with amitraz, suggesting that the mutation provides an evolutionary advantage in mites subjected to the selection pressure of the acaricide.

These variations are likely to disturb the correct folding of the protein, reduce the stability of the receptor, and affect the interaction with its ligand, but further work is needed to demonstrate the actual effect of such mutations in the normal function of the receptors and in their interactions with amitraz.

According to Marsky et al. [97], the development of amitraz resistance likely involves additional genetic variants and evolutionary processes, making the detection of resistance through markers more complex than simply identifying the presence of a single-nucleotide polymorphism.

Additionally, recent studies suggest that amitraz resistance tends to emerge in localized “patches” or “islands,” rather than uniformly affecting all honey bee colonies within an apiary or rapidly spreading across larger geographic areas [31,97].

Despite the growing interest on the amitraz genetic resistance mechanism [62,96,97], the genetic mechanisms underlying amitraz resistance in *V. destructor* are still unknown.

#### 3.4.3. Metabolic Resistance

Only two studies explored metabolic detoxification as a potential mechanism of resistance to amitraz. These studies analyzed esterase enzyme activity [22,38], as well as cytochrome P450 monooxygenase and glutathione-S-transferase activities [22]. However, no significant evidence of metabolic detoxification as a resistance mechanism was identified in either study.

Sammataro et al. [38] and Roth et al. [22] adopted a different methodological approach consisting in using, in different experimental settings, previously published discriminant concentration values as reference concentrations for the bioassay of mites. In detail, Sammataro et al. [38] used the discriminant concentration researched by Elzen et al. [28], while Roth et al. [22] used a discriminant concentration that was twice its reported LD90 by Sammataro et al. [38].

### 3.5. Risk of Bias Within Studies (Quality Evaluation)

The quality assessment of the included studies is summarized in Supplementary File S4. Papers achieving the maximum score of 5, such as Maggi et al. [33] and Marsky et al. [97], serve as exemplary benchmarks, showcasing well-documented methodologies and robust data reporting and underscore the critical importance of documented protocols.

Although the definition of ‘dead mite’ is the most frequently reported qualitative data (26 out of 33 studies), it is also the most variable in terms of interpretation and application. Group numerosity and number of replicates, along with the environmental parameters (T° and RH%) during the laboratory test, are essential for ensuring the reproducibility and robustness of data in scientific research, yet they are reported in fewer than 70% of the selected studies.

A clear indication of the months of mite collection was reported only in 19 papers. Correction for natural mortality was present only in 11 papers.

## 4. Discussion

Amitraz resistance in *V. destructor* populations is an increasing concern for apiculture worldwide, as evidenced by growing reports of treatment failures and declining efficacy in the field [97]. Monitoring and managing this resistance are critical to maintaining effective control of Varroa mites, which remain a significant threat to honey bee health. As recommended by Maggi et al. [29], beekeepers should assess mite populations both before and after miticide treatments to evaluate efficacy. Laboratory bioassays offer a controlled environment for precisely measuring amitraz resistance.

Bioassays designed to quantify the susceptibility of the Varroa mite to contact and volatile substances were developed by Milani [30] and Milani and Della Vedova [130], with detailed descriptions provided in Dietemann et al. [131]. While these methods have been outlined, they require updating and validation to ensure their continued relevance.

Establishing baseline LC50 values and dose–response curves for susceptible Varroa populations is essential for comparative analysis across time and regions [31] and, if genetic or biochemical mechanisms are still unknown, represent the most accurate and reliable method for comparing miticide resistance across Varroa populations [31,33].

Such baselines, standardized protocols, and harmonized experimental settings are critical to producing meaningful and consistent data.

The extreme variability in mortality observed during laboratory efficacy tests highlights the inconsistency in experimental methodologies across studies, making it nearly impossible to ensure cross-study comparisons and reliability.

The number of mites tested and the number of replicates, along with details on the control group, should be clearly documented [131,132].

It is essential to always specify the sample size in laboratory bioassays. Bioassays should include at least three replicates for each concentration, with at least ten Varroa mites per replicate, alongside a negative control group to validate the results. Furthermore, the control group must be used not only to exclude procedural errors, but also to accurately assess natural mortality.

Standardization of experimental protocols is particularly important because laboratory assays can vary significantly due to differences in mite strains and geographic locations and sampling periods, experimental design, and result elaboration.

The geographic diversity of mite populations and honey bee colonies provides valuable insights into how environmental and regional factors may influence treatment efficacy, as well as the physiological conditions of both mites and bees. This diversity includes differences in the developmental stages and physiological states of both Varroa mites and bees.

Certainly, laboratory susceptibility tests should account for the lipophilic properties of amitraz, as detailed in the *COLOSS Beebook* [131]. A proven approach involves incorporating amitraz into paraffin that coats the inner surface of test capsules [30,97,130].

The glass vial method, which involves coating the inner surface of vials with pesticides, ensures the uniform distribution of acaricide on the glass surface [23]; this method aligns with the recommendations of the Insecticide Resistance Action Committee (IRAC), but could cause high natural mortality rates [129]. To address high natural mortality rates caused by the glass vial method, plastic containers have been proposed as an alternative. For plastic materials, polypropylene or polyethylene are recommended to avoid the release of per- and polyfluoroalkyl substances (PFAS) which could interfere with experimental outcomes.

Contact could be either direct, as in glass vial assays, or indirect, as seen in apiarium setups; Baherini et al. [84] found that direct exposure to amitraz in glass vials resulted in nearly double the mite mortality (64%) compared to indirect exposure in apiarium (34%) after four hours. The experimental designs aimed to simulate natural conditions in which mites encounter treatments within hives. In laboratory settings, however, continuous exposure due to confined spaces may have heightened mite mortality and impacted bee behavior and stress, ultimately affecting their survival rates. For the type of contact, there is still no consensus; however, the method that most closely simulates the conditions of a.i. release in an apiary field setting should be preferred.

Additionally, it is essential to clearly specify the pre-setting conditions and management, such as the absence of recent queen introductions or merging with colonies previously treated with amitraz. The health of the colonies is also a pivotal factor, and it is crucial to report the absence of significant diseases as well as the overall robustness of the colonies’ nutritional status.

Amitraz exhibits relatively low lethality in bioassays, mainly due to its sublethal mode of action [28]. However, natural mite mortality must be accounted for in experiments. High mortality levels require attention to methodological factors such as environmental conditions (incubator temperature), mite origin, and timing of mite collection [94]. Different authors [22,28,38] further emphasize that experimental design, mite strain, and geographical location can significantly influence bioassay outcomes.

The acute toxicity of amitraz greatly varies between studies because mite bioassays are influenced by a variety of factors, which may include experimental design, mite strains, and geographical location; therefore, it is important to have a baseline value. It is crucial to better understand the impact of laboratory environmental and setting conditions on the test to enhance its reliability and reproducibility.

In order to detect emerging resistance in a mite population, the mechanisms of resistance should be known and target genetic analyses are needed [25].

For amitraz, establishing a baseline LC50 and dose–response curve in susceptible *V. destructor* populations is the most rigorous and reliable method for comparing miticide resistance across different Varroa populations. These populations can be tested either simultaneously from different regions or within the same region at different time points.

Once the discriminant dose has been determined, the target population could be tested. It is essential to recalibrate the discriminant dose annually, as resistance levels can fluctuate both between years and throughout the active season [97].

All laboratory efficacy studies can serve as baseline or comparisons for subsequent research to assess changes in resistance levels over time and across different locations if the same methodologies are repeated consistently.

Field studies, although essential, are complicated by factors such as environmental variables, apiary management practices, and other pests and diseases that can confound results. Laboratory assays mitigate these challenges by isolating variables, providing a clearer picture of acaricide efficacy.

Recently, a strong correlation between field efficacy tests and laboratory bioassays have been found, but these two testing methods should not be considered entirely interchangeable as measurements of resistance. Instead, their correlation provides a valuable framework for assessing and monitoring resistance development, offering insights that can help predict treatment failure with greater accuracy [31].

A consistent decline in efficacy below established thresholds should prompt deeper investigation into potential resistance and the exploration of alternative control strategies. It is crucial to recognize that reduced treatment efficacy is not solely attributable to pharmacoresistance. Various factors, including colony health, climatic conditions, and management practices, can significantly impact treatment outcomes, underscoring the need for a comprehensive evaluation of all possible contributing elements.

Uncovering the molecular mechanisms behind amitraz resistance is pivotal for early detection, predictions of treatment efficiency, and effective management strategies. The early detection of resistance, even before widespread treatment failures occur, is crucial to mitigating colony losses and preventing the spread of resistant mite strains [25,27].

The genetic mechanisms underlying amitraz resistance in *V. destructor*, as well as the metabolic detoxification processes, are only now beginning to be discovered and studied. Therefore, at the present moment, the resistance status of a Varroa population can only be confirmed by bioassays with a direct exposure of mites to the amitraz and amitraz-based products.

Despite this extended exposure, the field development of amitraz resistance appears to progress at a slower rate compared to other hard miticides [73].

Amitraz resistance monitoring presents significant challenges due to the high inter-colony variation within apiaries, where each colony may function as an isolated “island of resistance” with its own distinct Varroa population rather than uniformly affecting all honey bee colonies within an apiary or across regions [31,97].

To date, few studies [49,82,94,97] have investigated amitraz resistance by analyzing individual colonies within a single apiary, providing a more granular perspective on resistance distribution. All other research has pooled mites collected from different hives within the same apiary, treating the apiary as a single unit, or between different apiaries. This aggregation masks potential variability among colonies, potentially overlooking pockets of resistance localized to specific hives. The colony-specific approach offers a clearer understanding of resistance dynamics, emphasizing the importance of refining methodologies to better capture the heterogeneity within apiaries.

Additionally, according to EMA guidelines [132], the application of the product or active substance(s) should span multiple reproductive cycles of the parasite to assess the development and rate of resistance, as in Rodriguez-Hernandez et al. [95].

Therefore, enhancing the understanding of the prevalence, intensity, levels, trends, mechanisms, and genetics of amitraz resistance is crucial for developing effective strategies on a global scale. This effort must consider resistance dynamics both at the colony level and the potential dilutive effect when assessing resistance across the entire apiary, given the limited availability of effective active ingredients.

To address these challenges, national programs for recording treatment efficacy and monitoring resistance outbreaks should be established as part of broader pharmacovigilance efforts [49,133]. These programs would enable the timely reporting of inefficacy or resistance and guide the development of alternative control strategies. Monitoring the prevalence and trends in resistance is also crucial to avoid applying treatments that exert undue selection pressure, potentially exacerbating resistance. Furthermore, this approach could prevent or at least mitigate the disastrous impact that the tau-fluvalinate resistance crisis recorded in the 1990s had on the beekeeping industry [30].

Finally, as acaricides continue to be a cornerstone of Varroa management, expanding research to include other parasitic mites such as Acarapis and Tropilaelaps is increasingly important. Understanding amitraz resistance in these species will be critical for the global management of honey bee health.

## 5. Conclusions

It is of critical importance to understand how variations in test parameters influence the outcomes of the amitraz resistance tests, since the outcomes and interpretation of the amitraz resistance tests affect the application of the results to make effective *V. destructor* management decisions.

Resistance to amitraz is sometimes confirmed through laboratory testing, while in other cases, reports merely indicate reduced efficacy observed in the field or under experimental conditions. Laboratory studies are indispensable for accurately assessing resistance, as they provide a controlled environment that minimizes external variables and can reliably predict treatment effectiveness.

To comprehensively determine resistance status, a combination of laboratory testing, field observations, and molecular analyses is essential. Understanding the prevalence, intensity, mechanisms, and genetic basis of amitraz resistance is critically important, especially given the limited availability of effective active ingredients, veterinary medicinal products, and parasiticides.

The sustainable and efficient control of *V. destructor* mites in *A. mellifera* honey bee colonies continue to present significant challenges for beekeepers worldwide. Managing amitraz resistance effectively requires a multifaceted approach that integrates field-based insights, laboratory bioassays, advanced molecular diagnostics, and targeted policy interventions. By leveraging these complementary strategies, beekeepers, researchers, and policymakers can collaboratively work toward protecting honey bee populations and ensuring the long-term sustainability of apiculture.

## Figures and Tables

**Table 1:** Amitraz and amitraz-based product efficacy and resistance test conducted on bee-parasitic mite species on field and laboratory conditions.

Mite Species	Efficacy Laboratory Study	Efficacy Field Study	Resistance Assessment
*V. destructor* *	27 [28,31,32,33,38,48,49,54,61,80,81,82,83,84,85,86,87,88,89,90,91,92,93,94,95,96,97]	43 [28,37,48,50,60,62,69,71,72,74,80,85,97,98,99,100,101,102,103,104,105,106,107,108,109,110,111,112,113,114,115,116,117,118,119,120,121,122,123,124,125,126,127]	12 [22,29,31,38,51,62,73,95,96,97,128,129]
*T. clareae* and *V. destructor* * (coinfestation)		1 [70]	
*T. mercedesae*		1 [79]	
*A. woodi*	1 [78]	4 [75,76,77,78]	

Note: * named *V. jacobsoni* [28,69,70,71,72,73,74]. The first number = number of studies; [] = references.

**Table 2:** Result of laboratory efficacy test.

Commercial Miticidal Products	Method	Contact Time (Exposure)	Efficacy Assessment Duration	Dose	Mortality (%)	References
amitraz	Petri dish	1 h	1 h	0.4 μg/mL	61%	[80]
	Petri dish	1 h	1 h	0.4 μg/mL	64%	
	Petri dish	1 h	1 h	0.4 μg/mL	72%	
	glass vial	4 h	24 h	≥10 mg/L	≥90%	[83] *
	microapplicator	not applicable	24 h	≥0.000117 μg/mite	≥68%	
	glass vial	24 h	4 h	3.91 mg	65%	[84]
	glass vial	24 h	4 h	0.391 mg	47%	
	glass vial	24 h	4 h	0.0319 mg	58%	
	glass vial	24 h	24 h	3.91 mg	100%	
	glass vial	24 h	24 h	0.391 mg	89%	
	glass vial	24 h	24 h	0.0319 mg	60%	
	apiarium	4 h	4 h	3.91 mg	34%	
	apiarium	4 h	4 h	0.391 mg	27%	
	apiarium	4 h	4 h	0.0319 mg	10%	
	apiarium	4 h	24 h	3.91 mg	96%	
	apiarium	4 h	24 h	0.391 mg	82%	
	apiarium	4 h	24 h	0.0319 mg	31%	
	Petri dish	1 h	1 h	0.5 mg/mL	60%	[86]
	Petri dish	1 h	1 h	1 mg/mL	57%	
	Petri dish	1 h	1 h	2 mg/mL	100%	
	Petri dish	1 h	1 h	0.5 mg/mL	60%	[87]
	Petri dish	1 h	1 h	1 mg/mL	67%	
	Petri dish	1 h	1 h	2 mg/mL	93%	
	Petri dish	1 h	1 h	0.125 mg/mL	46%	[88]
	Petri dish	1 h	1 h	0.25 mg/mL	52%	
	Petri dish	1 h	1 h	0.5 mg/mL	51%	
	Petri dish	1 h	1 h	1 mg/mL	63%	
	Petri dish	1 h	1 h	2 mg/mL	90%	
	vial	24 h	24 h	0.09 mg/L	90%	[89]
	vial	24 h	24 h	0.0034 mg/L	10%	[90]
	vial	24 h	24 h	0.02 mg/L	50%	
	Petri dish	24 h	24 h	0.12 μg	64%	[33] *
	Petri dish	24 h	24 h	0.25 μg	72%	
	Petri dish	24 h	24 h	0.5 μg	92%	
	Petri dish	24 h	24 h	1 μg	100%	
	not specified	3, 6 and 24 h	3 h	100 mM	33%	[93] *
	not specified	3, 6 and 24 h	6 h	100 mM	40%	
	not specified	3, 6 and 24 h	24 h	100 mM	80%	
	Petri dish	1 h	49 h	110 μg/g	50%	[94] ***
	Petri dish	1 h	24 h	0.46 μg/mL (0.039 μg/cm^2^)	50%	[49] **
	Petri dish	1 h	24 h	0.39 μg/mL (0.22 μg/cm^2^)	90%	
	Petri dish	24 h	24 h	0.4 μg/mL	79%	
	Petri dish	24 h	24 h	0.4 μg/mL	75%	
	Petri dish	24 h	24 h	0.4 μg/mL	84%	
	Petri dish	24 h	24 h	0.4 μg/mL	77%	
	Paraffin capsules	1 h	24 h	2020: 28 ppm	90	[97] **
	Paraffin capsules	1 h	24 h	2021: 25 ppm	90	
	Paraffin capsules	1 h	24 h	28 ppm	89%	
	Paraffin capsules	1 h	24 h	28 ppm	78%	
	Paraffin capsules	1 h	24 h	28 ppm	73%	
	Paraffin capsules	1 h	24 h	28 ppm	95%	
	Paraffin capsules	1 h	24 h	28 ppm	98%	
	Paraffin capsules	1 h	24 h	28 ppm	74%	
	Paraffin capsules	1 h	24 h	28 ppm	77%	
	Paraffin capsules	1 h	24 h	28 ppm	100%	
	Paraffin capsules	1 h	24 h	28 ppm	94%	
	Paraffin capsules	1 h	24 h	28 ppm	91%	
	Paraffin capsules	1 h	24 h	28 ppm	100%	
	Paraffin capsules	1 h	24 h	28 ppm	96%	
	Paraffin capsules	1 h	24 h	28 ppm	100%	
	Paraffin capsules	1 h	24 h	28 ppm	96%	
	Paraffin capsules	1 h	24 h	28 ppm	97%	
	Paraffin capsules	1 h	24 h	28 ppm	98%	
	Paraffin capsules	1 h	24 h	28 ppm	100%	
	Paraffin capsules	1 h	24 h	25 ppm	93%	
	Paraffin capsules	1 h	24 h	25 ppm	87%	
	Paraffin capsules	1 h	24 h	25 ppm	68%	
	Paraffin capsules	1 h	24 h	25 ppm	98%	
	Paraffin capsules	1 h	24 h	25 ppm	92%	
	Paraffin capsules	1 h	24 h	25 ppm	81%	
	Paraffin capsules	1 h	24 h	25 ppm	85%	
	Paraffin capsules	1 h	24 h	25 ppm	95%	
	Paraffin capsules	1 h	24 h	25 ppm	91%	
	Paraffin capsules	1 h	24 h	25 ppm	1%	
	Paraffin capsules	1 h	24 h	25 ppm	66	
	Paraffin capsules	1 h	24 h	25 ppm	71%	
	Paraffin capsules	1 h	24 h	25 ppm	60%	
	Paraffin capsules	1 h	24 h	25 ppm	59%	
	Paraffin capsules	1 h	24 h	25 ppm	36%	
	Paraffin capsules	1 h	24 h	25 ppm	75%	
	Paraffin capsules	1 h	24 h	25 ppm	83%	
	Paraffin capsules	1 h	24 h	25 ppm	88%	
	Paraffin capsules	1 h	24 h	25 ppm	86%	
	Paraffin capsules	1 h	24 h	25 ppm	64%	
	Paraffin capsules	1 h	24 h	25 ppm	44%	
	Paraffin capsules	1 h	24 h	25 ppm	79%	
	Paraffin capsules	1 h	24 h	25 ppm	75%	
	Paraffin capsules	1 h	24 h	25 ppm	69%	
	Paraffin capsules	1 h	24 h	25 ppm	48%	
	glass scintillation vial	24 h	24 h	2.16 mg	10%	[28]
	glass scintillation vial	24 h	24 h	16.35 mg	50%	
	glass scintillation vial	24 h	24 h	123.51 mg	90%	
	glass scintillation vial	24 h	24 h	85.4 μg/mL	90%	
	glass scintillation vial	24 h	24 h	32.3 μg/mL	90%	
	glass scintillation vial	24 h	24 h	248 μg/vial	80–100%	[22] ****
	glass scintillation vial	24 h	24 h	123 micrograms	>90%	[38] *****
	Petri dish	1 h	48 h	141 μg/g	50	[81] *
	Petri dish	1 h	48 h	12 μg/g	50	
Apitraz^®^	mason jar	6 h	6 h	10.42 mg	89%	[82]
	mason jar	6 h	24 h	10.42 mg	100%	
	Petri dish	1 h	4 h	2.2 mg/cm^2^	100%	[61]
	Petri dish	1 h	24 h	2.2 mg/cm^2^	100%	
	Petri dish	1 h	4 h	2.1 mg/cm^2^	74% (2018)	[54]
	Petri dish	1 h	4 h	2.1 mg/cm^2^	81% (2019)	
	Petri dish	1 h	4 h	2.1 mg/cm^2^	92%	[95]
	Petri dish	1 h	4 h	2.1 mg/cm^2^	92%	
	Petri dish	1 h	4 h	2.1 mg/cm^2^	85%	
	Petri dish	1 h	4 h	2.1 mg/cm^2^	74%	
	Petri dish	1 h	4 h	2.1 mg/cm^2^	79%	
	Petri dish	1 h	4 h	2.1 mg/cm^2^	87%	
	Petri dish	1 h	4 h	2.1 mg/cm^2^	90%	
	Petri dish	1 h	4 h	2.1 mg/cm^2^	88%	
	Petri dish	1 h	24 h	2.1 mg/cm^2^	81%	
	Petri dish	1 h	24 h	2.1 mg/cm^2^	65%	
	Petri dish	1 h	24 h	2.1 mg/cm^2^	72%	
	Petri dish	1 h	24 h	2.1 mg/cm^2^	73%	
	Petri dish	1 h	24 h	2.1 mg/cm^2^	85%	
	Petri dish	1 h	24 h	2.1 mg/cm^2^	64%	
	Petri dish	1 h	24 h	2.1 mg/cm^2^	82%	
	Petri dish	1 h	24 h	2.1 mg/cm^2^	70%	
Apivar^®^	plastic cup	3 h	3 h	4 × 4 cm	100%	[31]
	apiarium	4 h and 24 h	4 h	24.15 mg	20%	[85]
	mason jar	4 h and 24 h	4 h	24.15 mg	17%	
	apiarium	4 h and 24 h	24 h	24.15 mg	68%	
	mason jar	4 h and 24 h	24 h	24.15 mg	58%	
	apiarium	4 h and 24 h	4 h	24.15 mg	48%	
	apiarium	4 h and 24 h	4 h	48.3 mg	43%	
	apiarium	4 h and 24 h	4 h	72.45 mg	49%	
	apiarium	4 h and 24 h	4 h	96.6 mg	45%	
	apiarium	4 h and 24 h	4 h	120.75 mg	53%	
	apiarium	4 h and 24 h	24 h	24.15 mg	76%	
	apiarium	4 h and 24 h	24 h	48.3 mg	63%	
	apiarium	4 h and 24 h	24 h	72.45 mg	71%	
	apiarium	4 h and 24 h	24 h	96.6 mg	51%	
	apiarium	4 h and 24 h	24 h	120.75 mg	54%	
	laboratory cage	48 h	6 h	2.76 g of strip	92%	[48]
	laboratory cage	48 h	48 h	2.76 g of strip	98%	
	mason jar	24 h	24 h	12.5 mg	>92%	[91]
	plastic container	3 h	24 h	0.008 μg	50%	[32] *
	plastic container	3 and 6 h	3 h	56.25 mg	99%	[93]
	plastic container	3 and 6 h	6 h	56.25 mg	100%	
	Petri dish	1 h	4 h	3.1 mg/cm^2^	2019: 79%	[54]
Mitaban^®^	glass vial	4 h	24 h	≥1000 mg/L	≥32%	[83] *
Taktic^®^	Petri dish	24 h	24 h	0.23 mg/L	50%	[92]
	microapplicator	not applicable	24 h	1.7 mg/L	90%	

Note: * Abbott’s formula; ** Schneider-Orelli’s formula; *** corrected for natural mortality; ***** acaricide amounts were set to produce approximately 90% mortality in susceptible mite populations [28,56]. **** These concentrations were twice their reported LD90 [33].

**Table 3:** Details on Varroa quantitative resistance assessment for amitraz and amitraz-based products.

Product	Investigated Population	Susceptible Population	Quantitative Resistance Assessment	References
amitraz	Czechia 2014	Czechia 2014	RR: 31.3, 7.5, 5.1	[51]
	Argentina 2009	Argentina 2007	RI: 3.9, 3.5, 3.7	[29]
	USA 2019	USA 2019	RR: 1.7, 3.9, 6.6, 2.6, 22.5 9.5 13.2, 7.9, 6.2, 3.2, 1.7	[31]
Taktic^®^	Mexico 2004	Mexico 2000	RI: 2.3, 12.77, 8.56, 26.66	[129]
	Mexico 2006–2007	Mexico 2000	RI: 12.77, 8.56, 26.66	[128]
Apivar^®^	France 1995	France 1998	MLT: 24.9 min (1995); 57.6 min, 45.5 min, 37.8 min (1998)	[73]

Note: RR = resistance ratio, RI = resistance index, MLT = mean lethal time.

## Data Availability

The data presented in this study are available within this article.

## Supplementary Materials

The following supporting information can be downloaded at: https://www.mdpi.com/article/10.3390/insects16030234/s1: Supplementary File S1 (Search strategy details); Supplementary File S2 (PRISMA flowchart diagram); Supplementary File S3 (Relevant data retrieved form laboratory and field test in elected articles) and Supplementary File S4 (Quality assessment).
