# Supplementary material for: Sensitivity and Resistance of Parasitic Mites (Varroa destructor, Tropilaelaps spp. and Acarapis woodi) Against Amitraz and Amitraz-Based Product Treatment: A Systematic Review
Source: Insects. 2025 Feb 20;16(3):234. doi: 10.3390/insects16030234 (PMC11942636; doi:10.3390/insects16030234)
Supplement: Supplementary file 1 [file insects-16-00234-s001.zip › Supplementary file S2.pdf]

PRISMA 2020 flow diagram for new systematic reviews which included searches of databases and registers only

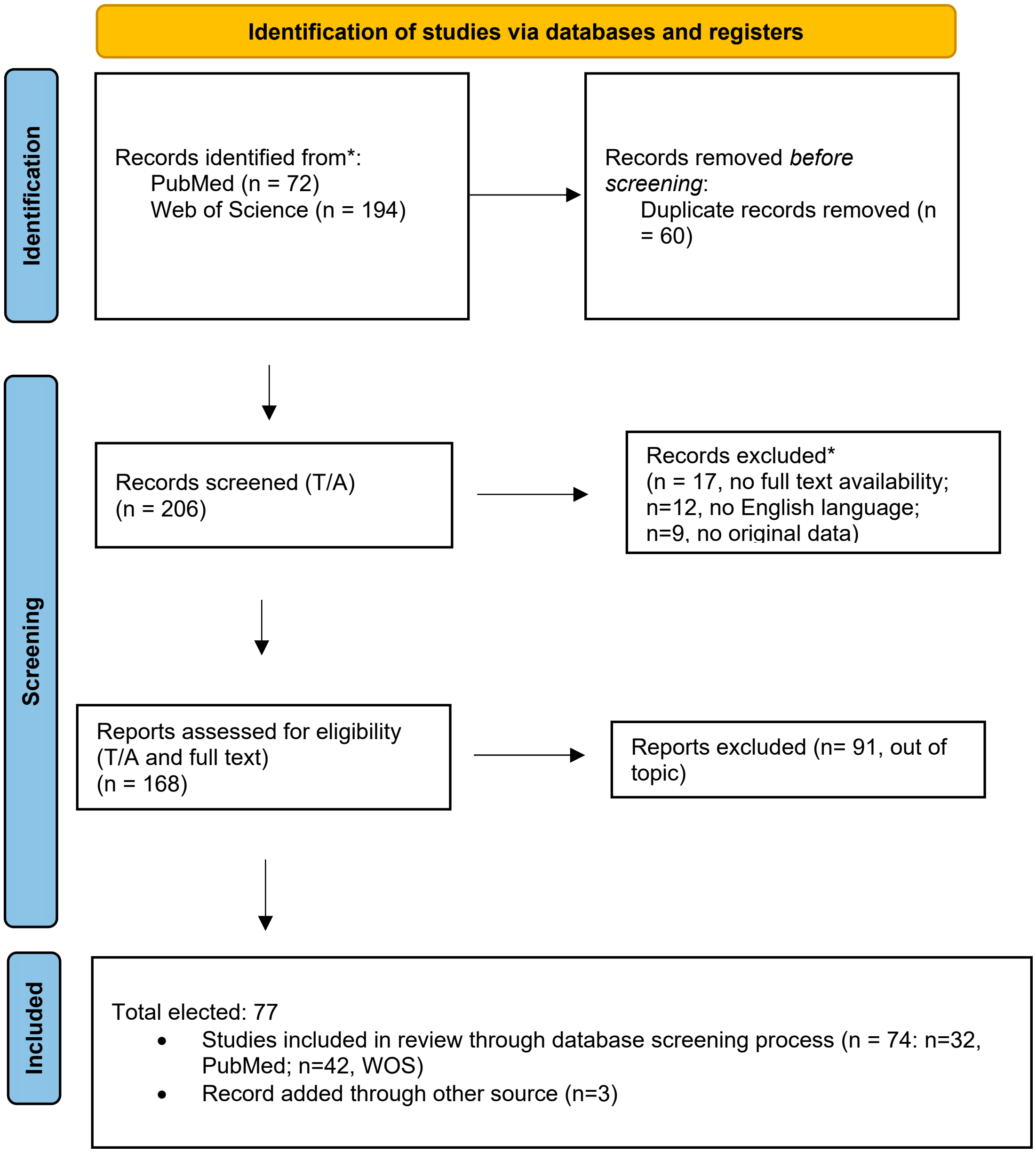

* Records were excluded by humans and not by automation tools.

From: Page MJ, McKenzie JE, Bossuyt PM, Boutron I, Hoffmann TC, Mulrow CD, et al. The PRISMA 2020 statement: an updated guideline for reporting systematic reviews. BMJ 2021;372:n71. doi: 10.1136/bmj.n71
